# Reduced receptor for advanced glycation end products is associated with α-SMA expression in patients with idiopathic pulmonary fibrosis and mice

**DOI:** 10.1186/s42826-021-00105-0

**Published:** 2021-10-02

**Authors:** Hyosin Baek, Soojin Jang, Jaehyun Park, Jimin Jang, Jooyeon Lee, Seok-Ho Hong, Woo Jin Kim, Sung-Min Park, Se-Ran Yang

**Affiliations:** 1 Department of Thoracic and Cardiovascular Surgery, School of Medicine, Kangwon National University, 1 Kangwondaehak-gil, Chuncheon, Gangwon 24341 Republic of Korea; 2 Department of Internal Medicine, School of Medicine, Kangwon National University, Chuncheon, Gangwon 24341 Republic of Korea

**Keywords:** IPF, RAGE, α-SMA, Fibrosis, Alveolar epithelial cells

## Abstract

**Background:**

Idiopathic pulmonary fibrosis (IPF) is a chronic and progressive interstitial lung disease. Despite alveolar epithelial cells is crucial role in lung, its contribution and the associated biomarker remain unknown in the pathogenesis of IPF. Recently, environmental factors including stone dust, silica and cigarette smoking were found as risk factors involved in IPF. Receptor for advanced glycation end products (RAGE) is a member of the immunoglobulin super family of cell surface receptors. It has been shown that interaction between RAGE and its ligands on immune cells mediates cellular migration and regulation of pro-inflammation. RAGE is highly expressed in the lung, in particular, alveolar epithelial cells. Therefore, we determined whether RAGE expression is associated with fibrosis-associated genes in patients with IPF and mice.

**Results:**

When bleomycin (BLM) was intratracheally administered to C57BL/6 mice for 1, 2 weeks, macrophage and neutrophils were significantly increased. The fibrotic nodule formed and accumulation of collagen was determined after BLM injection in H&E- and Masson’s trichrome staining. Levels of elastin, Col1a1 and fibronectin were increased in quantitative real-time PCR and protein levels of α-SMA was increased in western blot analysis. In the lung tissues of 1 mg/kg BLM-induced mice, RAGE expression was gradually decreased in 1- and 2 weeks in immunohistochemistry and western blot analysis, and 3 mg/kg of BLM-induced mice exhibited decreased RAGE levels while α-SMA expression was increased. We next determined RAGE expression in the lungs of IPF patients using immunohistochemistry. As a result, RAGE expression was decreased, while α-SMA expression was increased compared with non-IPF subjects.

**Conclusions:**

Our findings suggest that reduced RAGE was associated with increased fibrotic genes in BLM-induced mice and patients with IPF. Therefore, RAGE could be applied with a biomarker for prognosis and diagnosis in the pathogenesis of IPF.

## Background

Idiopathic pulmonary fibrosis (IPF) including symptom of dry cough and shortness of breath, is a chronic interstitial lung disease that occurs in people over 50 years old. Patients with IPF exhibited alveolar collapse, infiltration of immune cells, and accumulation of extracellular matrix [1]. Alveolar epithelial cell injury is a major feature in the pathogenesis of IPF [2]. Environment factor including stone dust or gas, silica and cigarette smoking exposures are considered as possible disease triggers in IPF progress [3]. Although IPF leads to symptoms of progressive accumulation of fibrotic tissues and its limited life expectancy of 2–5 years, the exact pathogenic mechanism remains elusive.

Receptor for advanced glycation end products (RAGE), encoded by the AGER gene, is a member of immunoglobulin super family of cell surface receptors [4]. RAGE activation is mediated by its ligand such as S100 protein [5], high-mobility group box 1 protein (HMGB1) [6] and advanced glycation end products (AGE) [7, 8]. The activated RAGE stimulates inflammatory signaling and then consequently leads to acute- and chronic inflammation. RAGE expression is abundant in the lung tissue, in particular, alveolar type 1 epithelial cells (AT1 cells) are highly expressed in the alveolar epithelium [9].

RAGE is expressed in three compositions containing one V-type domain, capable of binding ligands, and followed by two C-type domain in extracellular region [10]. In addition, the RAGE has three variant structures: (1) the full-length RAGE, (2) N-truncated type, and (3) C-truncated type which is secreted extracellularly [11]. Due to its different properties, it is critical to understanding a role of RAGE in various respiratory diseases. Soluble RAGE is found in serum and bronchoalveolar fluid (BALF), and produced by proteolytic cleaved from full-length RAGE that bound membrane [12]. In respiratory diseases, RAGE is significantly associated with many inflammation-related pathological states [13, 14]. Therefore, we determined distribution of RAGE in the mouse lung and patients with IPF. Moreover, we investigated how RAGE expression is associated with fibrotic genes in the pathogenesis of IPF.

## Results

### Increased inflammatory infiltrates accompanied by fibrotic changes in BLM-induced pulmonary fibrosis in mice

**Fig. 1:**
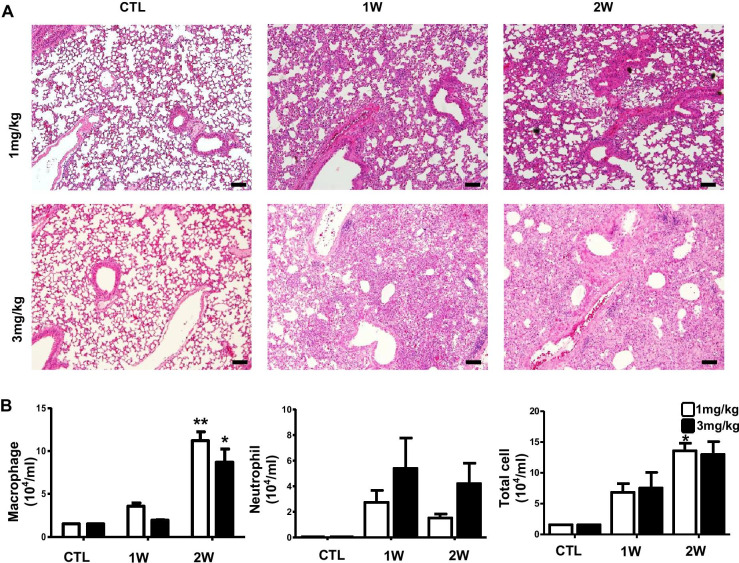
Increased neutrophils and macrophages infiltrate in BLM-induced pulmonary fibrosis in mice. Mice were injected intratracheally BLM (1, 3 mg/kg) for 1 and 2 weeks. **a** Represented images showed lung fibrosis by H&E staining. **b** Macrophages and neutrophils were counted using Wright-Giemsa staining image of mice BAL cells (n = 2 per group). Macrophages were increased in 1 and 2 weeks. Neutrophils were dramatically increased in 1 week that injected BLM 3 mg/kg. Scale bar: 100-μm. *p < 0.05 and **p < 0.01 compared with CTL. IPF, idiopathic pulmonary fibrosis; BLM, bleomycin; CTL, non-treatment

**Fig. 2:**
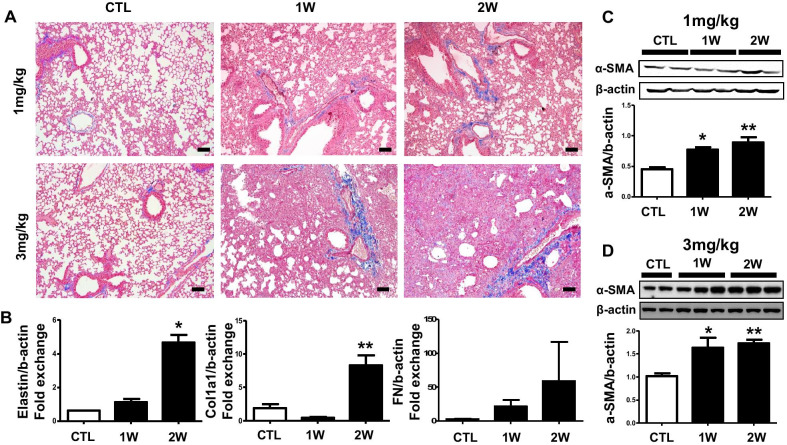
Increased inflammatory infiltrates accompanied by fibrotic changes in BLM-induced pulmonary fibrosis in mice. Mice were intratracheally injected BLM (1, 3 mg/kg) for 1 and 2 weeks. **a** Represented images showed collagen accumulation of lung by Masson’s trichrome staining. **b** The mRNA relative level of fibrosis marker was confirmed by real-time quantitative PCR. Western blot conclusion analysis determined accumulation of α-SMA in the **c** 1 ml/kg and (D) 3 ml/kg BLM treated lung. The intensity was quantified by Image J software. *p < 0.05 and **p < 0.01 compared with CTL

After intratracheally treated with two doses of BLM (1, 3 mg/kg) for 1 and 2 weeks, representative photomicrographs in H&E staining are exhibited (Fig. 1a). Histopathological assessment showed that mice represented diffuse changes consisting of combination of thickened alveolar septa, intra-alveolar fibrosis, and infiltration of neutrophils and macrophages. Total and differential cell count in BALF were determined on days 7 and 14 after the instillation of 1 and 3 mg/kg BLM. Number of both macrophages and neutrophils were significantly increased for 2 weeks (Fig. 1b). Lung hyperplasia by injury were increased immune cells and were caused structure change in mice lung. In order to elucidate characterization of IPF, we next assessed the Masson’s trichrome staining, real-time PCR and western blot analysis for fibrosis progression in the BLM-induced pulmonary fibrosis in mice. In Masson’s trichrome staining, we confirmed obvious histologic changes including excessive collagen accumulation, alveolar wall thickening, and neutrophil infiltration (Fig. 2a). Consistent with results in Masson’s trichrome staining, BLM administration significantly increased the mRNA levels of elastin, collagen1a1 and fibronectin (Fig. 2b). Compared to saline treated mice, protein level of α-SMA was up-regulated for 2 weeks in BLM treated mice (Fig. 2c, d).

### RAGE expression is down-regulated in BLM mouse model of pulmonary fibrosis

**Fig. 3:**
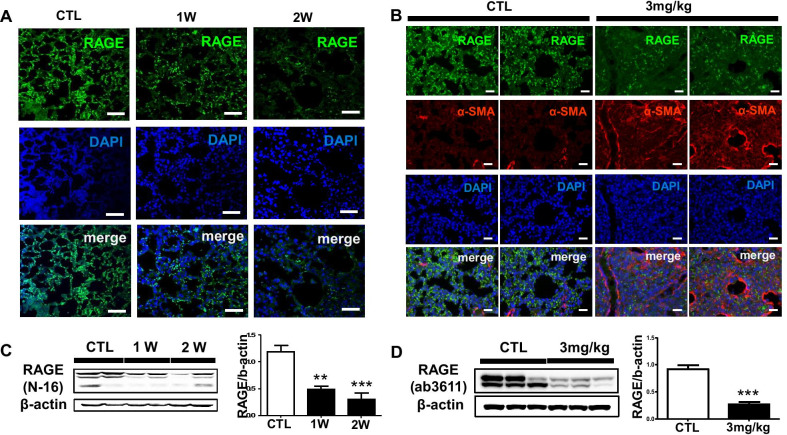
RAGE expression is down-regulated in BLM mouse model of pulmonary fibrosis. **a** The fluorescence expression of RAGE injured by BLM (1 mg/kg) for 1 and 2 weeks was captured by confocal laser microscopy. The chosen field were randomly obtained in X400 magnification. DAPI stained nuclei. Scale bar: 50-μm; **b** The fluorescence expression of RAGE and α-SMA injured by BLM (3 mg/kg) for 2 week was captured by EVOS M5000 imaging system. Scale bar: 200-μm; The Western blotting conclusion was shown RAGE expression affected by **c** 1 mg/kg BLM treated lung and **d** 3 mg/kg BLM treated lung for 2 weeks. The intensity was quantified by Image J software. **p < 0.01 and ***p < 0.001 compared with CTL

We next analyzed whether expression and distribution of RAGE in BLM-induced mice. In immunohistochemistry, RAGE expression was gradually decreased in the lung of 1 and 3 mg/kg BLM instillation (Fig. 3a). Since it has been demonstrated the role of RAGE—collagen interaction, we determined whether α-SMA expression is associated with RAGE expression after BLM injury. As shown in Fig. 3a, b, RAGE expression was gradually decreased in alveolar- and bronchial epithelium while α-SMA expression was enhanced in the wider area of the connective tissue of bronchial walls and thickened alveolar septa of the mouse lungs. That is, this is a result of demonstrating that the expression of RAGE in lung epithelial cells decreases by the structural change mechanisms of the lungs. In addition, we examined protein level of RAGE in mouse lung homogenates. RAGE expression was decreased in 1 and 3 mg/kg BLM exposed mouse lung for 1 and 2 weeks (Fig. 3c, d). Taken together, RAGE expression was decreased at the protein level after 1 and 2 weeks, while α-SMA expression was increased in mouse lung tissues.

### RAGE and α-SMA expression are differentially regulated in lung tissue of IPF patients

**Fig. 4:**
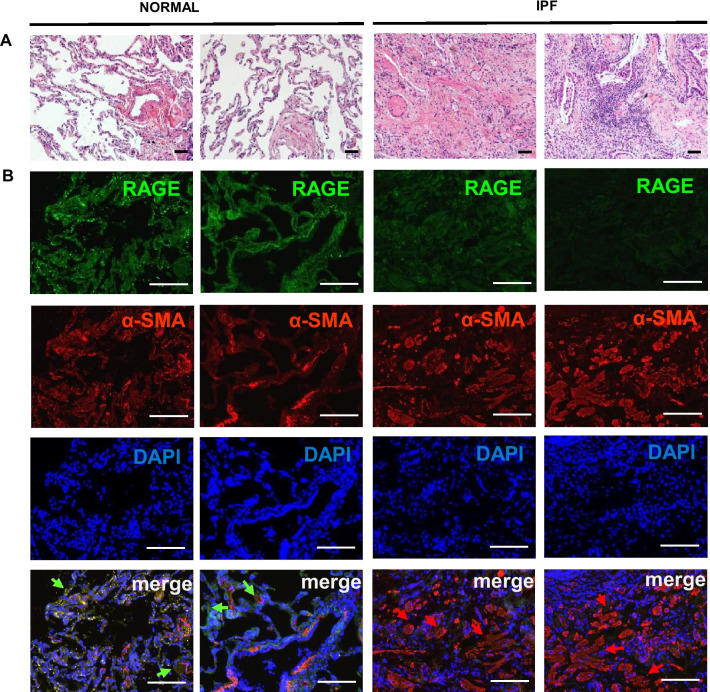
RAGE and α-SMA expression are differentially regulated in lung tissue of IPF patients. The represented images showed RAGE expression and collagen accumulation of IPF patient lung tissue. **a** H&E staining images of IPF patient lung tissue showed architecture damages. **b** Expression of RAGE and α-SMA were stained by immunohistochemistry. Green arrows were represented RAGE overexpression and Red arrows were represented α-SMA overexpression. **a** Scale bar: 50-μm; **b** Scale bar: 200-μm

We next determined the distributions of RAGE and α-SMA expression in donor and IPF patient lung tissue. IPF patients exhibited diffuse alveolar damage including thickening of alveolar septa, extensive airspace filling by macrophages compared to donor lung tissues (Fig. 4a). In immunohistochemistry, RAGE expression is decreased while α-SMA expression is widely observed in the connective tissue of bronchial walls, thickened alveolar septa, and bronchioles (Fig. 4b). These data are consistent with the observation shown on RAGE and α-SMA levels in the lungs of BLM-induced mice.

## Discussion

To recapitulate aspects of IPF disease, various animal models have been reported in various studies. Silica-induced pulmonary fibrosis disease has the advantage that silica particles could remain with persistent stimuli in the lung, [15]. FITC-induced method is replicable for fibrotic deposition by immunofluorescence staining. Moreover, fluorescent emission in FITC system allows observation fibrotic foci during fibrosis progression [16]. In addition, pro-inflammatory cytokines including TGF-β, TNF-α, IL-1β and IL-13 overexpression based techniques promote fibrotic signaling in the lung. However, this cytokine-mediated method is suitable for acute phase of inflammation rather than BLM-induced fibrosis model [17]. Even there are number of methods to induce and trigger pulmonary fibrosis, however it is not enough to explain clinical treatment of IPF. Hence, we decided to apply with BLM-induced pulmonary fibrosis model. It has been shown that therapeutic effects or anti-inflammatory responses using drugs or small molecules were confirmed in BLM-induced model [18–20]. In other words, BLM-induced model is helpful to validate therapeutic effect of drug [21].

In the lung, repeated alveolar epithelial cell damage causes to trigger progression of IPF [22]. In this regard, alveolar epithelial cell is crucial component in terms of lung homeostasis maintenance. Because alveolar epithelial cell has diverse roles, such as gas exchange, barrier function to defend the tissue from noxious factors and immunomodulatory function, these cells are critical component in terms of pulmonary homeostasis [23]. These cells most express RAGE in the lung, which is strongly associated with IPF pathogenesis [24]. RAGE pathway has been reported lung alveolar epithelial wound healing through cell migration and proliferation enhancement in vitro [25]. The RAGE is expressed and distinctly regulated in respiratory diseases. In previous study, the results have showed that IPF patients and asbestos-induced pulmonary fibrosis mice were decreased RAGE expression and RAGE-null mice enhanced fibrotic change [26]. These results support our arguments in perspective of between RAGE and IPF. In chronic respiratory diseases unlike IPF, PPE-induced chronic obstruct pulmonary disease (COPD) model has been reported that RAGE expression blockade has therapeutic effect [27]. Contrary to our results that RAGE expression were decreased in IPF including chronic disease tendency, the protein expression up-regulated in COPD. In acute respiratory diseases, RAGE expression was significantly overexpressed in acute lung injury/acute respiratory distress syndrome (ALI/ARDS) [28, 29]. In acute lung disease progression, overexpression of the RAGE, which has a crucial influence on the maintenance of lung structural, is a major pathogenesis of these diseases [30]. Moreover, in these acute model, the RAGE expression were increased and the disease were accompanied neutrophil infiltration and lung permeability, edema formation, and apoptosis [29].

Thus, the RAGE is differentially expressed and characterized in various diseases. Queisser et al. reported that blockade of RAGE with siRNA-mediated RAGE knockdown differentially inhibited cellular adhesion, migration and proliferation in A549 cells and pulmonary fibroblasts. They suggested that siRNA knockdown of RAGE in A549 cells affected mainly proliferation, whereas in pulmonary fibroblasts elevated cell migration. Consistent with Queisser’s results, our findings demonstrate that loss of RAGE is associated with increased fibrotic genes in BLM-induced mice and IPF patients, suggesting that RAGE plays a key role in regulation of cellular adhesion and integrin function.

Previously, we have shown that RAGE is a major receptor to induce sustained inflammation and alveolar epithelial injury in the pathogenesis of COPD [27]. Our results suggest that continuous loss of alveolar epithelial cells and increased fibroblasts via promoted ECM accumulation leads to down-regulation of RAGE during IPF progression. In summary, we found that decreased expression of RAGE is associated with loss of alveolar epithelial cells, and the increased fibrotic and inflammatory cells failed to remove and replace injury of alveolar epithelial cells in IPF. Therefore, prevention of reduced RAGE in alveolar epithelial cells may provide protective mechanisms and accurate diagnosis of IPF.

## Conclusions

In this study, our results suggest the implication of RAGE down-regulation in the pathogenesis of IPF. The expression of RAGE in the BLM-induced pulmonary fibrosis decreased while fibrosis progression increased. In particular, RAGE distribution was decreased in IPF lung tissues as well as IPF experimental mouse model. Taken together, the advance in the knowledge of RAGE and its involved mechanisms in IPF would be essential to investigate in the lung homeostasis and aging.

## Methods

### Materials

Bleomycin (European Pharmacopoeia Reference Standard, B1141000) was purchased from Sigma-Aldrich (St. Louis, MO, USA). Primary antibodies against β-actin (#3700) was purchased from Cell Signaling Technology Inc. (Beverly, MA, USA), α-SMA (ab5694) and RAGE (ab3611) was purchased from Abcam (Abcam, Cambridge, UK), and RAGE (A-9) (sc-365694), and RAGE (N-16) (sc8230) were purchased from the Santa Cruz Biotechnology, Inc (Santa Cruz, CA, USA).

### Animals

C57BL/6 male mice (7–8-week-old, 20–23 g) were obtained from DooYeol Biotech (Seoul, Korea) and were reviewed by Institutional Animal Care and Use Committee (No. KW-180903-1) approval. For Idiopathic pulmonary fibrosis (IPF) animal model, WT mice were intratracheally injected to 1 mg/kg, 3 mg/kg of BLM (bleomycin) in saline. The mice were sacrificed on 1 or 2 week after BLM injection, and the lung were harvested and stored at -80 °C immediately for further analysis.

### Human lung sample preparation

The collection of human samples was approved from the ethics committee of Kangwon National University Hospital in Korea (IRB NO. KNUH-2014-10-015). The lung tissue samples used as donor or IPF were obtained from human non-malignant 4 lung tissue specimens of lung cancer patient or patients undergoing diagnostic surgical lung biopsy for IPF. All provided by the Soonchunhyang University Hospital of National Biobank and Pusan National University Hospital of National Biobank (n = 4). RAGE and α-SMA in lung tissues were determined by IHC.

### Lung tissue histology

On 1, 2 weeks after BLM injection, mice were sacrificed and left lung were inflated by 4% paraformaldehyde for at least 24 h then lung embedded in paraffin. The paraffin block was cut 5 µm section for staining with H&E for histological analysis.

### Bronchoalveolar lavage fluid cell staining

Bronchoalveolar lavage fluid (BALF) was obtained with 18-gauge intravascular catheter and 1 ml saline through trachea. The obtained BALF was centrifuged at 3000 rpm for 10 min, and then pellets were resuspended in 1 ml saline after the supernatant was removed. For BAL cell transfer on slide, only 100μL of resuspended cell was cyto-centrifuged at 3000 rpm for 10 min. The slides were fixed and stained by HEMA 3 staining pack (Fisher Scientific Co LLC, MI, USA, 123-869) according to instruction manual.

### Western blot analysis

All proteins from human and mouse lung tissues were extracted by RIPA lysis buffer supplemented with two protease inhibitor cocktail (GenDEPOT, TX, USA; P3100, P3200). For isolate 25 μg soluble protein, protein was quantified with Bicinchoninic (BCA) Protein Assay Kit (Thermo Scientific, MA, USA; 23225). Then, proteins were separated by 10–15% SDS-PAGE, the sample was transferred to membrane 0.45 μm (BIO-RAD, CA, USA; 162–0115) and membranes were blocked with 5% skim milk in TBST with 0.05% Tween 20 (MBcell, CA, USA; MB-S1667). Immunoblotting was performed using the following primary antibodies with 1:1000–10,000 diluted in 1 × TBST for overnight at 4°C. After 3times wash for 10 min, the membranes were probed with polyclonal anti-rabbit/mouse or goat HRP-conjugated secondary antibodies for 1 h at room temperature and then detected using ECL solution. The membrane was scanned using the ChemiDOC™ imaging system (Bio-Rad Laboratories, Hercules, CA, USA).

### Masson’s trichrome staining

The Masson’s trichrome staining was used for measurement of collagen deposition in lung tissue. The lungs for staining were fixed 4% formaldehyde, embedded in paraffin and cut into 6 μm sections. Slides were stained using MTS kit’s (American MasterTech) and images were captured using microscope.

### Quantitative real-time PCR

The RNA from mouse lung tissue was extracted using Trizol reagent (#79306; Qiagen, NRW, Düsseldorf, Germany) according to manufacturer’s protocol. For cDNA synthesis, 1 μg of total RNA was carried out reverse transcription-polymerase chain reaction in each tube using Revers Transcription Master Premix (#EBT-1514; Elpis Biotech, Daejeon, Korea). And then real-time PCR was performed using a Step One Plus real-time PCR system (Applied Biosystems, Warrington, UK) with SYBR Green (#RT501; Enzynomics, Daejeon, Korea). Relative mRNA expression was normalized against β-actin expression along 2 − ΔΔCt methods. The used primers sequences in this study were represented in Table 1.

**Table 1:** Primer list

Genes	Sequences	NM number
Elastin	F-GGGTCTGACAGCGGTAGTC	NM_007925.4
	R-TGGAGGAGGTTGAGCAAGA	
Collagen, type 1, alpha 1	F-TGGAAGAGCGGAGAGTACTG	NM_007742.4
	R-TGATGGCGTCCAGGTT	
Fibronectin	F-CCATGAAGCAACGTGTTATG	NM_010233.2
	R-TCTGCCACTGTTCTCCTACAT	
β-actin	F-AGGCCAACCGTGAAAAGATG	NM_007393.5
	R-CACAGCCTGGATGGCTACGT	

The primers were used to confirm fibrosis progression by quantitative real-time PCR

### Immunohistochemistry

Paraffin-embedded tissues were cut into 5 μm slices and mounted on slides. After deparaffinization and rehydration step, hidden epitope between paraformaldehyde and protein appeared by antigen retrieval buffer. The slides were blocked in peroxidase-blocking solution for 20 min and 10% normal goat serum for 1 h and primary antibody stained with anti-RAGE (1:500) and anti-α-SMA (1:500) overnight. The sectioned tissues were secondary antibody stained with Alexa Fluor 488 goat anti-mouse (Invitrogen, A11001, 1:2000) and Alexa Fluor 488 goat anti-rabbit (Invitrogen, A11012, 1:2000) for 1 h at room temperature. Finally, slides were mounted with DAPI mounting solution and were detected using an LSM 510 confocal laser-scanning microscope and EVOS M5000 imaging system (M5000, Thermo fisher, USA).

#### Statistical analysis

The results were represented as the mean ± sem. Oneway-ANOVA followed by Bonferroni's Multiple Comparison Test was used for comparisons between multiple groups. A value of *P* < 0.05 was considered statistically significant using Graph Pad prism 5 program to calculate.

## Data Availability

Not applicable.
